# Psychometric Properties of the Personal Report of Public Speaking Anxiety (PRPSA) in a Sample of University Students in Sweden

**DOI:** 10.1007/s41811-018-0022-0

**Published:** 2018-09-27

**Authors:** Ewa Mörtberg, Markus Jansson-Fröjmark, Axel Pettersson, Tove Hennlid-Oredsson

**Affiliations:** 1 Department of Psychology, Stockholm University, SE-106 91 Stockholm, Sweden; 2 Department of Clinical Neuroscience, Karolinska Institute, Stockholm, Sweden; 3 Centre for Psychiatry Research, Department of Clinical Neuroscience, Karolinska Institute, Stockholm, Sweden

**Keywords:** Fear of public speaking, Personal Report of Public Speaking Anxiety (PRPSA), Psychometric properties

## Abstract

Existing measures for examining fear of public speaking are somewhat limited in content and there is a need for scales that assess a broader area including cognitive, behavioral, and physiological dimensions of the fear. This study examined the psychometric properties of the Personal Report of Public Speaking Anxiety (PRPSA) in a sample of university students (*n*, 273). Participants completed the PRPSA and measures of depression, social and general anxiety, and quality of life. A reduced version of the PRPSA, the PRPSA-18, was found to demonstrate satisfactory internal consistency as well as discriminant and convergent validity. The PRPSA-18 was associated with two solid factors, “Anticipatory anxiety and physiological symptoms during speech performance,” and “Lack of control during speech performance.” A PRPSA-18 score of 58 was found to discriminate participants with higher and lower fear of public speaking. It is concluded that the shorter and more easily administered PRPSA-18 is a credible option for assessing fear of public speaking among university students.

## Introduction

Fear of speaking or performing in front of an audience, i.e., performance anxiety, is common in the general population. It has been estimated to 60–77% in social anxiety disorder (SAD; Diagnostic and Statistical Manual-5/DSM American Psychological Association/APA 2013) populations (Stein et al. 2000; Furmark et al. 1999), and to 14–25% in the absence of SAD in normal populations (Furmark et al. 1999; Kessler et al. 1998; Wittchen and Fehm 2003). Performance anxiety could be a part of the diagnosis of SAD, which implicates fear of being negatively scrutinized in social performance and social interaction situations, which typically are associated with excessive anxiety and avoidance of the feared situations and a clinically significant impairment of social and occupational functioning. In the recent DSM-5 (APA 2013), performance anxiety is a specifier of SAD. This specific type of SAD is in comparison with the (abovementioned) “typical” SAD regarded as a milder expression of the disorder. Some authors (e.g., Pull 2012) have, however, suggested that it rather may be an isolated and qualitatively different subtype of SAD. It is, for example, associated with stronger physiological reactions (e.g., panic attacks) during speech performance, is typically not associated with comorbid psychiatric conditions or innate behavioral inhibition, and has usually a later onset (about 17 years of age) (Blöte et al. 2009; Bögels et al. 2010). Also, sub-clinical performance anxiety, which is common in the population (e.g., Furmark et al. 1999), is reported to be associated with high anxiety and considerable distress and impairment as well as negative personal and economic consequences (e.g., Blöte et al. 2009).

Within student populations, the reported prevalence estimates of SAD are 11.6 (Baptista et al. 2012) and 16.1% (Tillfors and Furmark 2007), which is approximately consistent with those found in general populations (Ruscio et al. 2008). In contrast to the more severe expression of SAD typically found in the general population, performance anxiety appears to be more prevalent in student populations. For example, Tillfors and Furmark (2007) found that the most common fear among Swedish students (*n*, 523) was fear of speaking or performing in front of an audience. Some 71% of students with a diagnosis of SAD (*n*, 84) reported this fear. Also, a considerable proportion of students without SAD (8.7%) reported performance anxiety (Tillfors and Furmark 2007). However, an even higher proportion of students with sub-clinical performance anxiety (24.2%) was found by Baptista et al. (2012) in a study of Brazilian students (*n*, 2319).

As reported by Russel and Shaw (2009), social anxiety could have detrimental effects for academic performance with failures in examinations and graduation, and a negative impact on future career development. Thus, it is of great importance to identify not only individuals with SAD but also individuals with sub-clinical levels of fear of public speaking, who may be at risk for a negative development. However, self-report scales for identifying and assessing fear of public speaking in particular are rather limited. Most existing scales focus on cognitive aspects of fear of public speaking, e.g., the Speech Anxiety Thoughts Inventory (Cho et al. 2004) and the Self-Statements During Public Speaking (Hofmann and Dibartolo 2000), whereas the Public Speaking Anxiety Scale (PSAS; Bartholomay and Houlihan 2016; Gilkinson 1942) and the Personal Report of Public Speaking Anxiety (PRPSA; McCroskey 1970) also cover behavioral and physiological aspects of social anxiety. A recent study of the PSAS reported satisfactory psychometric properties of the scale (Bartholomay and Houlihan 2016). One difference between the PSAS and the PRPSA is that the PRPSA more directly addresses fear of public speaking in educational settings. Since performance anxiety is highly prevalent in student populations, the PRPSA could be particularly useful in such contexts. The PRPSA would potentially benefit research and clinical settings by providing a psychometrically sound instrument that can be used in cross-sectional and experimental research, in diagnosing fear of public speaking, and in evaluating the treatment of this fear in student populations.

The psychometric properties of the PRPSA have been addressed in only two previous studies. The first study (McCroskey 1970) of a student sample (*n*, 769) found that the scale was unifactorial and associated with excellent internal consistency (0.94) and good test-retest reliability (0.84). The second study (Hsu 2012) in a Taiwanese student sample (*n*, 82) reported six factors of the PRPSA and excellent internal consistency (0.90). However, these studies did not examine the convergent and discriminant validity of the PRPSA. Also, the construct validity of the scale is still undetermined. McCroskey (1970) did not reveal any details of the factor analyses, and the sample size of Hsu’s study (2012) is too small for extracting meaningful factorial dimensions. Determining the factor structure of PRPSA would help to decide the number of relevant items that should belong to the scale. In its present composition, it may be too extensive (34 items) and could possibly be reduced.

Thus, in order to find an assessment that particularly captures fear of public speaking, the aim of the present study was to examine the factor structure and convergent and discriminant validity as well as the sensitivity and specificity of the PRPSA in a relatively large sample (*n*, 273) of university students in Sweden.

## Materials and Methods

### Participants

The participants of the study consisted of a combined sample of students from the National College of Defence and the Department of Psychology in Stockholm, Sweden (n: 273). In sample I (*n*, 112), 44.6% studied political science and the remaining participants studied the psychologist program. In sample II (*n*, 161), all participants studied psychology of which 79.5% attended a basic course in psychology and the remaining participants studied the psychologist program. These students received course credits for their participation. The average age of participants in the total sample was 26.9 years (SD 6.9, age range 19–51 years). In sample I, the mean age was 25.8 years (SD = 5.8, range 19–45 years) and in sample II 27.7 years (SD 7.5, range 20–51 years). In the total sample, 71.4% were women (sample I 65.2%, sample II 75.8%).

### Procedure

The study participants in sample I were asked during classes to participate in the study, and, if willing, completed the survey via paper and pencil. Sample II was contacted through an e-mail which informed about the possibility to participate in the study through a unique web platform. Participants in sample II completed the online published questionnaire during four weeks in 2016. All participants were informed that their questionnaires would be used for research, and that their participation was voluntary and could be discontinued at any time. All participants signed an informed consent.

### Measures

All participants (*n*, 273) completed self-reports of public speaking anxiety (PRPSA), whereas only sample II (*n*, 161) conducted self-reports of social anxiety, general anxiety, depression, and quality of life.

#### The Personal Report of Public Speaking Anxiety

PRPSA (McCroskey 1970) is a 34-item scale developed for measuring fear of public speaking. Each item is rated on a 5-item Likert scale ranging from 1 (*strongly disagree*) to 5 (*strongly agree*). Twenty-two items are negatively formulated, e.g., “I feel anxious while waiting to give my speech,” and 12 items are positively formulated, e.g., “I enjoy preparing for a speech.” In the analyses of these data, the positively formulated items of PRPSA were reversed so that high scores on the scale consistently reflected higher fear of public speaking. Cronbach’s alpha of the scale has been shown to range from 0.84 to 0.94 (McCroskey 1970; Hsu 2012). The PRPSA was translated to Swedish by two of the authors (AP and TH) and back translated to English by an authorized translator who was blind to the original scale. There were no inconsistencies between the translations.

#### The Social Phobia Inventory

The Social Phobia Inventory (SPIN; Connor et al. 2000; authorized Swedish translation by Svanborg, Hedman and Mörtberg) is a 17-item self-rated 0–4 Likert scale ranging from 0 (*not at all*) to 4 (*extremely*), which evaluate fear, avoidance, and physiological discomfort in a variety of social situations. The scale (with scores ranging between 0 and 68) has been reported to show good to excellent test-retest reliability, internal consistency, and convergent and divergent validity (Connor et al. 2000; Sosic et al. 2008). The internal consistency of the SPIN in the present sample was excellent (Cronbach’s *α* = 0.90). The cut-off scores for discriminating subjects with SAD from controls have been reported to differ between 19 and 25 points (Connor et al. 2000; Nagata et al. 2013; Sosic et al. 2008). Scores higher than 3 (*very much*) on item 11 (“I avoid having to give speeches”) was used in the receiver operating characteristics (ROC) analyses for deciding cut-off scores for the PRPSA. Item 11 was used since it explicitly determines the level of public speaking avoidance, thus making it useful as a marker of substantial fear of public speaking.

#### The Generalized Anxiety Disorder Screener

The Generalized Anxiety Disorder Screener (GAD-7; Spitzer et al. 2006) consists of seven items that assess anxiety-related symptoms, which the person might have experienced during the past two weeks, e.g., “Feeling nervous, anxious or on edge.” It is rated on a 4-point Likert scale ranging from 1 (*not at all*) to 4 (*nearly every day*). The GAD-7 is associated with satisfactory reliability (Cronbach’s *α* = 0.89–0.94) and convergent validity (Löwe et al. 2008; Mills et al. 2014). A GAD-7 score of > 10 have been reported to suggest an anxiety disorder (Löwe et al. 2008). The internal consistency of the scale in the present sample was excellent (Cronbach’s *α* = 0.90).

#### The Patient Health Questionnaire

The Patient Health Questionnaire (PHQ-9; Kroenke et al. 2001) is a screening measure of major depressive disorder (MDD; APA 1994). Nine items are rated on a 4-point Likert scale (0 *= not at all*; 3 *= nearly every day*) corresponding to the DSM-IV criteria for depression. The tenth item assesses functional level (“How difficult have these problems made it for you to do your work, take care of things at home, or get along with other people?”). A PHQ-9 score of ≥ 10 has shown a sensitivity and specificity of 88% for capturing MDD. The internal consistency of the PHQ-9 in the present sample was good (Cronbach’s *α* = 0.88).

#### Brunnsviken Brief Quality of Life Scale

The Brunnsviken Brief Quality of Life Scale (BBQ; Lindner et al. 2016) is a 12-item scale assessing the respondent’s satisfaction with quality of life within six central life areas, e.g., “Learning is important for my quality of life.” The Swedish standardization of the BBQ was based on 167 students (40 men and 127 women aged 26.9 years (SD 7.8). Lindner et al. (2016) have reported good test-retest reliability (0.89) and acceptable internal consistency (Cronbach’s *α* = 0.68). The internal consistency of the BBQ in the present sample was acceptable (Cronbach’s *α* = 0.74).

### Statistical Analyses

Version 22 of the SPSS statistical software was used for conducting all analyses. Exploratory factor analysis (EFA) according to recommendations by Costello and Osborne (2005) was applied for exploring the latent structure of the PRPSA-34. The Kaiser-Meyer-Olkin (KMO) measure of sampling adequacy and the Bartlett test of sphericity (BTS) were used in order to explore the factorability of the data. As the data set of the sample (*n*, 273) showed an approximately normal distribution, a maximum likelihood factoring method (Fabrigar et al. 1999) was used with oblique rotation (direct oblimin), which allows factors to correlate and was expected in this data set. The parallel analysis (O’Connor 2000) was a base for decision of how many factors should be retained for rotation. According to recommendations (Costello and Osborne 2005), items with factor loadings at minimum 0.30 after rotation, with no or few cross-loadings (0.32 or higher) on two or more factors, and with communalities above 0.40, were retained. A factor of five or more strongly loading items (.50 or more) is suggested to be a strong factor, whereas a factor consisting of less than three items is considered to be a weak factor (Costello and Osborne 2005).

Internal consistency of the PRPSA was examined by Cronbach’s *α*. Convergent and discriminant validity was examined by correlations of the PRPSA with the SPIN, the GAD-7, the PHQ-9, and the BBQ. To examine the accuracy of the PRPSA to discriminate cases with high and low fear of public speaking, ROC analyses were used to examine sensitivity and specificity of the scale in order to identify optimal cut-off scores. The area under the curve (AUC) describes the scales’ capability to discriminate high and low fear of public speaking. A case processing accuracy of AUC of 0.90 can be interpreted as “excellent,” 0.80–0.90 as “good,” 0.70–0.80 as “fair,” and 0.60–0.70 as “poor.”

One-way analysis of variance (ANOVA) was used to examine differences in PRPSA scores and scores of additional measures in participants with higher and lower fear of public speaking.

## Results

### Factor Structure of the PRPSA-34

The introductory analyses of the factorability of the PRPSA-34 indicated a KMO index of 0.97 and a significant BTS (*χ*^2^ (7159, 6), *p* < 0.001), which indicated that the data were suitable for factor analysis. The parallel analysis (O’Connor 2000) suggested two factors. A further exploration of this solution (explaining 53.3% of the variance) revealed several cross-loading items (items 3, 8, 10, 11, 12, 22, 27, 28, and 29) and items with low communalities (i.e., < .40; items 7, 13, 14, 15, 19, 23, and 25). Thus, in the further analyses, these items were gradually discarded in order to improve the factorial structure, and six of the reversed scored items were removed during these analyses. In the final solution (accounting for 60.4% of the variance), two stable factors including 18 items emerged (Table 1). Factor 1 was labeled “Anticipatory anxiety and physiological symptoms during speech performance,” and factor 2 “Lack of control during speech performance.”

**Table 1:** Factor loadings of the Personal Report of Public Speaking Anxiety in an exploratory factor analysis with maximum likelihood extraction and direct oblimin rotation

PRPSA items	Factor 1	Factor 2
1. While preparing for giving a speech, I feel tense and nervous.	*.74*	− .22
2. I feel tense when I see the words “speech” and “public speech” on a course outline when studying.	*.91*	− .27
5. I get anxious when I think about a speech coming up.	*.89*	.01
6. I have no fear of giving a speech.	*.69*	.09
9. When the instructor announces a speaking assignment in class, I can feel myself getting tense.	*.90*	−.06
18. I do not dread giving a speech.	*.66*	.16
20. My heart beats very fast just as I start a speech	*.44*	.23
21. I experience considerable anxiety while sitting in the room just before my speech starts.	*.60*	.21
26. I feel comfortable and relaxed in the hour or so just before giving a speech.	*.74*	− .07
31. I have trouble falling asleep the night before a speech.	*.71*	**−** .02
32. My heart beats very fast while I present a speech.	*.46*	.30
33. I feel anxious while waiting to give my speech.	*.86*	.02
4. Right after giving a speech I feel that I have had a pleasant experience.	− .10	*.71*
16. I feel that I am in complete possession of myself while giving a speech.	.10	*.76*
17. My mind is clear when giving a speech.	.09	*.75*
24. While giving a speech, I know I can control my feelings of tension and stress.	.10	*.66*
30. During an important speech I experience a feeling of helplessness building up inside me.	.29	*.53*
34. While giving a speech, I get so nervous I forget facts I really know.	.01	*.76*

Items 4, 6, 16–18, and 24 are reverse-scored items. Italicized numbers indicate primary loadings

### Internal Consistency

Excellent internal consistency was found for both the original 34-item PRPSA (Cronbach’s *α* = 0.97) and the reduced 18-item PRPSA (*α* = 0.96). The average inter-item correlation of the PRPSA-18 was 0.55.

### Discriminant and Convergent Validity of the PRPSA-18

Overall, there was a strong correlation (0.99) between the original PRPSA-34 and the PRPSA-18. In addition, the two PRPSA-18 factors were highly correlated (0.78).

Discriminant validity of the PRPSA-18 was indicated by the weak correlation with depressive symptoms (PHQ-9), and the weak negative correlation with quality of life (BBQ) (Table 2). Quality of life was overall significantly, but negatively, related to depressive symptoms, general anxiety (GAD-7), and social anxiety (SPIN), i.e., higher quality of life was related to lower levels of depression and anxiety*.* Convergent validity of PRPSA-18 was evidenced by the strong positive correlations with social anxiety (SPIN) and avoidance of public speaking (SPIN item 11), and the moderate correlations with general anxiety (GAD-7) and high social anxiety (> 25 points on the SPIN). Age was unrelated to the PRPSA-18, whereas gender (being a woman) was weakly, but significantly, correlated with the scale.

**Table 2:** Correlations between the Personal Report of Public Speaking-18 (PRPSA-18) and measures of anxiety, depression, and quality of life

	1	2	3	4	5	6	7	8	9	10	11
1. PRPSA-18	1										
2. PRPSA-18: factor 1	.97**	1									
3. PRPSA-18: factor 2	.88**	.75**	1								
4. SPIN item 11	.68**	.63**	.66**	1							
5. Avoid public speaking	.60**	.55**	.60**	.88**	1						
6. Social anxiety	.60**	.55**	.60**	.88**	.23**	1					
7. SPIN	.52**	.47**	.52**	.52**	.58**	.81**	1				
8. Age	− .02	.00	− .07	.10	.05	.05	.01	1			
9. Gender	.18*	.20*	.09	.07	.02	.13	.09	− .05	1		
10. GAD-7	.38**	.38**	.32**	.15*	.10	.38**	.42**	− .21**	.20*	1	
11. PHQ-9	.24**	.22**	.25**	.08	.01	.33**	.36**	− .22**	.14	.73**	1
12. BBQ	− .16*	− .11	− .24**	− .14	− .09	.31**	− .40**	.13	− .12	− .46**	− .57**

Avoid public speaking = > 3 (“very much” and “extremely”) on the SPIN item 11, BBQ = Brunnsviken Brief Quality of Life scale, Factor 1 = *Anticipatory anxiety and physiological symptoms during speech performance*, Factor 2 = *Lack of control during speech performance*, GAD-7 = Generalized Anxiety Disorder Screener, PHQ-9 = Patient Health Questionnaire, PRPSA = Personal Report of Public Speaking Anxiety, Social anxiety = high or low social anxiety based on the SPIN cut-off score, SPIN = Social Phobia Inventory, SPIN item 11 = “I avoid having to give speeches.” *p < .05; **p < .01

### Analyses of the SPIN in Relation to the PRPSA

An inspection of the SPIN item correlations with the PRPSA-18 showed that six SPIN items correlated weakly (0.1–0.3), one item was non-correlated, and nine items correlated moderately with the PRPSA-18. Only one item showed a strong correlation (0.68) to PRPSA-18 (item 11 “I avoid having to give speeches”). For the ROC analyses, scores of > 3 and < 3 on this item were used to classify “higher” and “lower” fear of public speaking.

### Classification Accuracy

The accuracy of the PRPSA-18 to discriminate participants with higher and lower fear of public speaking anxiety was good (0.86, 95% CI 0.80–0.91) as measured by the AUC. The confidence interval of the AUC value did not include 0.5, which indicates that the analysis could discriminate between participants with higher and lower fear of public speaking better than chance. A cut-off score of 58 points of the PRPSA-18 was found to demonstrate the most optimal sensitivity (0.86) and specificity (0.70).

### Higher Versus Lower Fear of Public Speaking (*n*, 161)

Based on the cut-off score of the PRPSA-18, 94 participants (78 women and 16 men; 58.4%) showed higher fear of public speaking (Table 3). These participants differed significantly from those with lower public speaking fear on social anxiety (SPIN) and general anxiety (GAD-7) and trended to (*p* = 0.053) higher rates of depressive symptoms (PHQ-9). Among those with higher public speaking fear, 28 participants (23 women and 5 men; 17.4%) scored above 25 points on the SPIN, which indicates SAD (e.g., Sosic et al. 2008). Thus, it appears that a considerable proportion of participants suffer from speaking fear in the absence of indicated SAD.

**Table 3:** Scores of the Personal Report of Public Speaking Anxiety-18 (PRPSA-18) among participants with higher and lower fear of public speaking

	Higher fear (*n*, 94)	Lower fear (*n*, 67)	Group effect [*F* (1, 160)]
	M (SD)	M (SD)	
PRPSA-18	70.9 (9.0)	44.6 (9.4)	320.9***
SPIN	20.6 (11.7)	12.9 (9.1)	20.6***
BBQ	36.6 (6.1)	37.5 (5.7)	0.8
GAD-7	7.9 (5.1)	4.5 (4.5)	19.9***
PHQ-9	7.9 (5.9)	6.1 (5.3)	3.8

“Higher” versus “lower” fear of public speaking was based on the PRPSA-18 above or below 58. BBQ = Brunnsviken Brief Quality of Life scale, GAD-7 = Generalized Anxiety Disorder Screener, PHQ-9 = Patient Health Questionnaire, PRPSA = Personal Report of Public Speaking Anxiety, SPIN = Social Phobia Inventory. ***p < .001

## Discussion

In this study, the psychometric properties of the Swedish PRPSA-34 were examined in a university student sample with the aim to explore the factorial structure, internal consistency, validity, and sensitivity and specificity of the scale. To summarize, the results indicated satisfactory psychometric properties of a reduced version, the PRPSA-18, supporting the use of this version of the scale for assessment of fear of public speaking among university students.

In contrast to previous studies, which have reported one (McCroskey 1970) or six (Hsu 2012) underlying factors of the PRPSA-36, two solid factors emerged of the PRPSA-18 in our study. The factor dimensions were associated with (1) anxiety prior to and during a public speech and (2) feelings of helplessness and lack of control during a speech. These dimensions of performance anxiety might reflect similar maintaining processes that have been reported for SAD. Anticipatory anxiety, e.g., “I have trouble falling asleep the night before a speech” (factor 1), is a common problem among individuals with social anxiety. According to the cognitive model developed by Clark and colleagues (Clark and Wells 1995; Clark 2001), anticipatory anxiety is related to specific cognitive and emotional processing prior to a feared event. The individual will carefully review what will happen in the situation and just thinking of the event will activate anxiety and associated thoughts dominated by memories of past failures, negative self-images, and predictions of future poor performance (Clark et al. 2001). High in-situation anxiety, e.g., “My heart beats very fast while I present a speech” (factor 1), is according to this model related to excessively high standards of social performance associated with thoughts of failure and negative evaluation. This will lead to increased anxiety and a negative self-focused attention, which, for example, can result in experiences of feeling blocked or helpless, e.g., “While giving a speech, I get so nervous I forget facts I really know” (factor 2).

In our two-factor solution, half of the reversed score items were omitted, which in addition to the advantage of a shorter administration is a further improvement. That is, reversed scored items may be confusing for the respondent and also impractical for the user to score. In some studies, reversed score items have been found to produce spurious factor structures (e.g., Rodebaugh et al. 2007), which, however, was not indicated in this study. McCroskey (1970) reported that the PRPSA-34 was unifactorial in a large student sample (*n*, 732) but the details of the factor analytic procedure were not reported and could, thus, not be discussed here. Hsu (2012) reported six factors of the scale, which appears less valid due to the small sample (*n*, 82) permitting a subject-to-item ratio of only a little bit over 2:1. Only 10% of studies with such subject-to-item ratio have been found to reach a correct factor structure, and increasing the ratios would result in increasingly correct factor structures (Costello and Osborne 2005). About 60% of studies with an item-to-subject ratio of 1:10, as in the present study, have been found to reach a correct factor structure (Costello and Osborne 2005). Thus, it would be of importance to further examine the PRPSA-18 in larger samples.

Consistent with previous studies (Hsu 2012; McCroskey 1970), we found that the PRPSA-34 was associated with excellent internal consistency, which was evident for the PRPSA-18 as well. The validity of the PRPSA has, however, not previously been reported. In the current study, divergent validity was verified by the weak correlations with depressive symptoms and quality of life. The convergent validity was evidenced by the strong-to-moderate positive relation between fear of public speaking, social anxiety (SPIN), and general anxiety (GAD-7). This may be expected as fear of public speaking is closely related to general social anxiety as well as a diagnosis of SAD.

Moreover, the accuracy of the PRPSA-18 to discriminate higher and lower fear of public speaking anxiety was good as measured by the ROC and the AUC and estimated to a cut-off score of 58. Most probably, a proportion of students in the current sample are suffering from SAD, indicated by the fact that some 17% (mostly women) of those who reported high fear of public speaking also had a score of 25 or above on the SPIN. In comparison, the prevalence estimates of SAD in the general populations are typically around 15% (e.g., Furmark et al. 1999), and similar prevalence estimates have been reported in university student populations (Tillfors and Furmark 2007; Baptista et al. 2012). However, in the present study, a considerably larger proportion of participants (39%) reported high fear of public speaking in the absence of indicated SAD. This estimation is substantially higher than previous discrepant reports, indicating 8.7% (Tillfors and Furmark 2007) and 24.2% (Baptista et al. 2012). However, the estimations in these studies were based on single items, i.e., “Speaking (or performing) in front of a group of people” (Tillfors and Furmark 2007) and “Avoids speeches” (Baptista et al. 2012), whereas our estimation was based on a range of items related to fear of public speaking. However, it is likely that the larger proportion of participants with social anxiety in our study is related to the convenience sampling method so that students prone to social anxiety were more inclined to participate. Also, participants were not formally diagnosed so the actual prevalence of SAD and sub-clinical fear of public speaking could not be validly determined.

Some limitations of the study need to be mentioned. First, some characteristics of the sample should be underscored. The use of relatively young university students might hamper the generalizability to other groups in the community and to patient populations. It should also be noted that a large majority of the study participants were women. A related limitation, which might hinder generalizability, is also that the sample used in this study was a convenience sample. A randomized selection of students from different universities in Sweden or adults from the general public would have been preferable. Second, participants were not formally diagnosed so we were unable to validly infer how individuals with clinical public speaking anxiety (and typical SAD) may score on the PRPSA-18. Third, the study lacks psychometric information concerning test-retest reliability and treatment sensitivity of the PRPSA-18. Despite these limitations, there are some notable strengths of the study. The strengths include the sound psychometric properties of the supplementary rating scales, the relatively large sample to enable information about some important psychometric properties of the PRPSA, and recruitment of students from different departments and levels.

To conclude, fear of public speaking and general social anxiety are very common in a selected student population. The PRPSA-18, which might reflect maintaining factors of the fear, is a reliable and valid measure for assessing fear of public speaking. It would potentially benefit research and clinical settings by providing a psychometrically sound instrument that may be used for diagnosing fear of public speaking as well as evaluating its treatment. As implicated above, studies in randomized selections of participants are needed for further exploration of the psychometric properties of the PRPSA among clinical and non-clinical subjects.
